# Vitamin C supplementation lowers advanced glycation end products (AGEs) and malondialdehyde (MDA) in patients with type 2 diabetes: A randomized, double‐blind, placebo‐controlled clinical trial

**DOI:** 10.1002/fsn3.3530

**Published:** 2023-06-30

**Authors:** Soghra Rabizadeh, Firouzeh Heidari, Reza Karimi, Armin Rajab, Shahram Rahimi‐Dehgolan, Amirhossein Yadegar, Fatemeh Mohammadi, Hossein Mirmiranpour, Alireza Esteghamati, Manouchehr Nakhjavani

**Affiliations:** ^1^ Endocrinology and Metabolism Research Center (EMRC) Vali‐Asr Hospital, Tehran University of Medical Sciences Tehran Iran

**Keywords:** clinical trial, diabetes, inflammation, oxidative stress, vitamin C

## Abstract

This study evaluated how daily vitamin C administration impacts systemic oxidative stress and inflammation and its safety in T2D patients. This randomized, double‐blinded, placebo‐controlled, parallel‐arm clinical trial included 70 patients with T2D. They were allocated to receive either 500 mg/day of vitamin C or a matching placebo for 8 weeks. Of the 70 subjects assigned to the trial, 57 were included in the statistical analysis (vitamin C: *n* = 32, placebo: *n* = 25). Inflammatory and oxidative markers, including advanced glycation end products (AGEs), malondialdehyde (MDA), advanced oxidation protein products (AOPP), oxidized low‐density lipoprotein (ox‐LDL), highly sensitive C‐reactive protein (hs‐CRP), tumor necrosis factor‐α (TNF‐α), and ferric reducing ability of plasma (FRAP) were measured at baseline and the end of the trial. In addition, vitamin C tolerance was evaluated. A nutritionist visited all participants for a standard diabetic regimen. Following vitamin C supplementation, the serum levels of MDA (*p*‐value < .001) and AGEs (*p*‐value = .002) demonstrated a significant decrease after controlling for multiple confounders, including age, blood pressure, waist circumference, HbA1C, TG, and LDL‐C, while no significant changes were observed for AOPP (*p*‐value = .234) and ox‐LDL (*p*‐value = .480). The FRAP showed an increasing trend as an antioxidant marker but was not statistically significant (*p*‐value = .312). The hs‐CRP and TNF‐α had no significant changes (*p*‐value: .899 and .454, respectively). Also, no major adverse events were observed. Vitamin C supplementation may be beneficial in reducing AGEs and MDA in patients with T2D.

## Graphical abstract

This study showed that adding 8 weeks of vitamin C supplementation to hypoglycemic medications had significant beneficial effects on MDA and AGEs, which are long‐lived molecules in patients with type 2 diabetes.

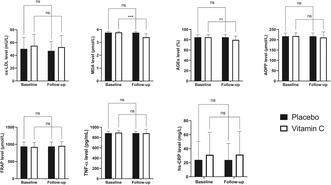

## 1 INTRODUCTION

In the twenty‐first century, diabetes is one of the leading causes of debility and death, with an ascending prevalence pattern (Hu, 2011; Wild et al., 2004). Diabetes predisposes patients to various complications, such as atherosclerosis, neuropathy, nephropathy, retinopathy, and myocardial infarction (American Diabetes Association, 2010; Yadegar et al., 2022). Due to the high mortality and morbidity rates, rigorous management should be provided for patients with diabetes (Spellman, 2003).

There is convincing evidence that oxidative stress has a critical role in the pathogenesis of type 2 diabetes (T2D) and the development of diabetes complications (Barman & Srinivasan, 2016; Faria & Persaud, 2017; Scott & King, 2004). Elevated blood glucose concentration leads to inappropriate production of reactive oxygen species (ROS), which are under the tight control of endogenous antioxidant defense mechanisms, including enzymatic and nonenzymatic pathways in healthy cells (Matough et al., 2012). In patients with diabetes, the function of the endogenous antioxidant defense system is impaired (Kowluru & Chan, 2007; Matough et al., 2012). Intensification of ROS production leads to endothelial dysfunction, impaired insulin secretion, impaired glucose disposal, and systemic inflammation (Heidari et al., 2019; Qahremani et al., 2022; Styskal et al., 2012). The increase of inflammatory cytokines provokes more production of ROS and creates a vicious cycle between oxidative and inflammatory mediators (Esposito et al., 2002; Evans et al., 2002). Modern diets are heat‐processed, linked to circulating advanced glycation end products (AGEs) in health and disease (Uribarri et al., 2010). They can increase oxidative stress and inflammation, especially in diabetes and cardiovascular disease, as well as diabetic nephropathy and cataract (Barman et al., 2018; Barman & Srinivasan, 2019; Liu et al., 2017; Pradeep et al., 2019; Thomas et al., 2005; Uribarri et al., 2015). Nutritional factors such as fruits, vegetables, and antioxidants affect redox status and may inhibit glycation (Vlassopoulos et al., 2014).

There is a growing interest in applying dietary micronutrients with antioxidative and anti‐inflammatory properties for patients with diabetes. It has been shown that ascorbic acid (vitamin C) can reduce insulin resistance and cardiovascular complications in patients with T2D (Badawi et al., 2010; Bartlett & Eperjesi, 2008). Vitamin C is an essential cellular antioxidant that detoxifies free radicals, regenerates circulating antioxidants, and scavenges ROS (Arrigoni & De Tullio, 2002; Mescic Macan et al., 2019).

Previous studies that evaluated the effects of vitamin C on oxidative stress (ox‐stress) and inflammation in T2D showed conflicting results. An analysis of the impact of vitamin C on the oxidative parameters in gestational diabetes showed a significant decrease in malondialdehyde (MDA) and an increase in glutathione and superoxide dismutase (SOD) (Maged et al., 2016). Another study observed a significant reduction in C‐reactive protein (CRP) and Interleukin‐6 (IL‐6) levels with vitamin C supplementation in patients with obesity, hypertension, and/or diabetes (Ellulu et al., 2015). In contrast, two clinical trials demonstrated that vitamin C supplementation did not affect oxidative stress in T2D (Darko et al., 2002; Tousoulis et al., 2007). Also, another study reported that the combination of vitamin C and vitamin E supplementation did not improve AGE levels in skin samples of patients with T2D (Konen et al., 2000).

The current study aimed to evaluate further the effect of daily administration of vitamin C on systemic oxidative stress and inflammatory activity and its safety in patients with T2D. Several serum markers of oxidative stress were measured, including AGEs, advanced oxidation protein products (AOPP), oxidized low‐density lipoprotein (Ox‐LDL), MDA, and ferritin reducing ability of plasma (FRAP). In addition, inflammatory activity was evaluated by measuring tumor necrosis factor‐α (TNF‐α) and highly sensitive C‐reactive protein (hs‐CRP).

## 2 MATERIALS AND METHODS

### 2.1 Study population

The sample size and power calculation were done with the appropriate sample size formula (Zhong, 2009) based on the previous study (Vinson & Howard III, 1996) (*α* = 0.05; *β* = 0.2). Participants aged 30–65 years with T2D based on the American diabetes association (ADA) criteria (American Diabetes Association, 2020) were enrolled in this study. This study screened 90 patients for eligibility.

Conditions that may act as cofounders in the study were excluded, including class III/IV heart failure, any history of vascular complications of diabetes or hypertension, any history of chronic disease (including lung, kidney, or liver disease), body mass index (BMI) more than 35 kg/m^2^, systolic blood pressure (SBP) more than 160 mmHg, diastolic blood pressure (DBP) more than 90 mmHg, use prescription or over‐the‐counter vitamins, smoking history, alcohol use in the past month, aspirin intake in the past year, with or planning pregnancy, history of corticosteroid intake, history of hypersensitivity to vitamin C, and history of acute or chronic inflammation (Appendix S1).

### 2.2 Study design

This study was an 8‐week, randomized, double‐blinded, placebo‐controlled, and parallel‐arm clinical trial conducted between October 2018 and March 2019 in outpatient diabetes clinics of Vali‐Asr Hospital, affiliated with Tehran University of Medical Sciences. Randomization was performed by a computer‐generated code, with an allocation ratio of 1:1. The assignment was conducted using sealed opaque envelopes. Vitamin C and placebo were identical in size, shape, color, and odor, which participants and outcome raters could not distinguish between the groups. In addition, research coordinators and medication dispensers were all blinded to the allocations. Eligible patients (*n* = 70) were randomized to receive 500 mg of vitamin C daily or a matching placebo added to their hypoglycemic medication. Medication adherence was controlled by counting the tablets consumed and comparing the calculated number with the report of medication intake by the patients. The exact nutritional schedule was considered for both groups, which focused on the same consumption of vegetables and fruits in all participants. Patients were also instructed to continue the lifestyle they had before the study. Patients were asked to inform the research team in case of any unexpected symptoms during the trial period. At the follow‐up visit, all participants were systematically assessed for any adverse effects during the trial, using open‐ended questions followed by a 25‐item checklist of side effects (Appendix  S2).

Before the commencement of the trial, the procedures and purpose were explained, and the participants gave written informed consent before enrollment. The protocol of the current study was in accordance with the Declaration of Helsinki and was approved by the Ethics Committee of the National Institute for Medical Research Development of Iran (NIMAD; IR.NIMAD.REC.1397.123). This trial was registered in the Iranian Registry of Clinical Trials (IRCT; https://www.irct.ir; Registration number: IRCT20160811029306N1).

### 2.3 Physical examinations

Trained staff performed examinations. The height measurement was made with rigid tape and a precision of 0.1 cm. Patients were asked to dress in light clothing while their weight was measured using a portable digital scale (Tefal PP1100). According to the Quetelet equation, the body mass index (BMI) was calculated by dividing weight (kg) by height squared (m^2^). The blood pressure was measured three times, 5 min apart, with calibrated Omron M7 digital sphygmomanometers (Hoofddorp, The Netherlands) and arm cuffs covering at least 80% of the arm. The mean blood pressure value was calculated using the second and third records. With the subject standing still on a flat surface, waist circumference (WC) was measured using a nonstretchable measuring tape. WC was measured midway between the costal margins and the iliac crest in the horizontal plane.

### 2.4 Laboratory evaluations

Venous blood samples were collected at baseline and after 8 weeks, between 08:00 a.m. and 09:00 a.m., after about 12 h of fasting. Then, each sample was centrifuged, and the serum was divided into multiple aliquots and kept in a freezer at −70°C until the biochemical analyses. Fasting plasma glucose (FPG) was measured by the glucose oxidase method (Parsazmun; Auto Analyzer, BT‐3000(plus), Biotechnica), and glycated hemoglobin A1c (HbA1c) by the high‐performance liquid chromatography (HPLC) method (DS5, DREW, England). Cholesterol, triglyceride (TG), low‐density lipoprotein cholesterol (LDL‐C), and high‐density lipoprotein cholesterol (HDL‐C), as well as urea, creatinine, and uric acid, were determined using enzymatic methods (Parsazmun; Auto Analyzer, BT‐3000(plus), Biotechnica). Serum alanine aminotransferase (ALT), aspartate aminotransferase (AST), and alkaline phosphatase (ALP) were assessed using enzymatic photometry (Parsazmun; Auto Analyzer, BT‐3000(plus), Biotechnica). Insulin was measured by the chemiluminescence method (Immunotech; IMMULITE 2500, SIEMENS). Homeostasis model assessment of insulin resistance (HOMA‐IR) was calculated as follows: HOMA‐IR = (FBS (mg/dL) × insulin (mu/L)/405) (Esteghamati et al., 2010). The Cockcroft‐gault formula ([140–age] × [weight per kg] × [0.85 if female])/(72 × [serum creatinine per mg/dL]) was used to estimate the Glomerular filtration rate (GFR) (Cockcroft & Gault, 1976). Serum oxidative and inflammatory biomarkers were also assessed.

### 2.5 Oxidative and inflammatory biomarkers

The primary outcome of this study was changes in the serum oxidative biomarkers, including AGEs, AOPP, MDA, and ox‐LDL from baseline by vitamin C during the 8 weeks of treatment. Secondary outcomes were changes in FRAP, as an antioxidant marker, and inflammatory markers, including TNF‐α and hs‐CRP.

Advanced glycation end products (AGEs) were measured using the spectrofluorimetric analysis described by Kalousova et al. (2002) (Shimadzu, RF‐5000). Serums were diluted in phosphate‐buffered saline (PBS) by a factor of 50. Fluorescence intensity was measured at 350 nm excitation and 440 nm emissions and expressed as a percentage of fluorescent emission. The intraassay and the interassay coefficient of variation were 5.1% and 7.9%, respectively.

Advanced oxidation protein products (AOPP) was assessed with the spectrophotometric method of Kalousova et al. (2002) (FLUOstar OPTIMA, BMG). A quantity of 200 μL of serum was diluted by a factor of 5 in PBS. In addition, for calibration, 200 μL of chloramine T (0–100 μmol/L) and 200 μL of PBS as a blank were added to different microplates. Finally, 10 μL of acetic acid and 20 μL of 1.16 M potassium iodide (KI) were added to the preparations. Measurements were performed at the absorbance of 340 nm and expressed in μmol/L. The intraassay and the interassay coefficient of variation were less than 5% and less than 10%, respectively.

As described by Benzie et al., FRAP was measured with spectrophotometry (Benzie & Strain, 1996) (Shimadzu, UV‐3100). This method prepared the FRAP reagent by mixing 300 mmol/L acetate buffer (pH:3.6) and 10 mmol/L tripyridyl triazine (TPTZ) in 40 mmol/L HCL and 20 mmol/L FeCl3.6H2O. Then, 25 μL of serum was added to 750 μL of FRAP reagent. Measurements were done at the absorbance of 593 nm and expressed in μmol/L. We plotted the FRAP calibration curve. Necessary dilution was made to ensure that the FRAP value was within the linear range of the standard curve. The mean and standard deviation were calculated. The intraassay and the interassay coefficient of variation were 3% and 4.2%, respectively.

Malondialdehyde (MDA) concentration was assessed using colorimetric method (μmol/L; Cayman; Mindray, MR‐96A). The intraassay and the interassay coefficient of variation were 5.5% and 5.9%, respectively.

Oxidized low‐density lipoprotein (Ox‐LDL) was assessed using a sandwich ELISA kit (Mercodia; Mindray, MR‐96A). The intra‐ and interassay coefficient of variation were 4% and 7.3%, respectively.

Samples were triplicated for AGEs, AOPP, and FRAP. If the concentration was above the calibration range, the samples were assessed with dilution, and the values were multiplied by the dilution coefficient. The measurement was repeated if the concentration was lower than the dynamic calibration range. All samples were assayed in duplicate.

TNF‐α serum levels were measured using an ELISA kit (Diaclone Besancon; Mindray, MR‐96A). The intra‐ and interassay coefficient of variation were 3.2% and 10.9%, respectively. Serum levels of hs‐CRP were assessed using a two‐site ELISA (Diagnostic Biochem; Mindray). The intra‐ and interassay coefficient of variation were 5% and 9.5%, respectively.

### 2.6 Statistical analysis

All analyses were performed on participants who completed the trial. Probability graphics and the Shapiro–Wilk test were used to test the compliance of variables with normal distribution. Quantitative and qualitative variables were expressed as mean, standard deviation (SD), and percentage, respectively. The baseline level of quantitative and qualitative variables between the intervention and placebo groups were compared using Student's *t*‐test and Chi‐square, respectively. Between‐ and within‐group comparisons were performed using independent samples and paired t‐tests. Fisher's exact test was used to compare adverse events between the groups. The effect size in terms of the raw mean difference was provided for each variable. The variables with nonnormal distribution were expressed as median (interquartile range) and compared between groups using the nonparametric Mann–Whitney *U*‐test. Univariate analysis of covariance (ANCOVA) was applied to evaluate the difference between the effectiveness of the two interventions. The measured markers of the primary outcome were entered as dependent variables, while possible confounding categories and baseline measurements were considered independent variables and covariates, respectively. The ANCOVA model assumes no strong correlation exists between the confounding variables (i.e., colinearity). Therefore, three variables, including HbA1C, HDL, and total cholesterol, were not entered into the same model.

Each analysis calculated the effect size from partial eta squared according to Cohen's recommendations (1%, 6%, and 13.8% were considered small, medium, and large effects, respectively). The SPSS package version 24.0 for Windows (IBM Corporation, New York, USA) was used for statistical analysis. A *p*‐value of lower than .05 was considered statistically significant for all tests.

## 3 RESULTS

### 3.1 Baseline characteristics

**FIGURE 1:**
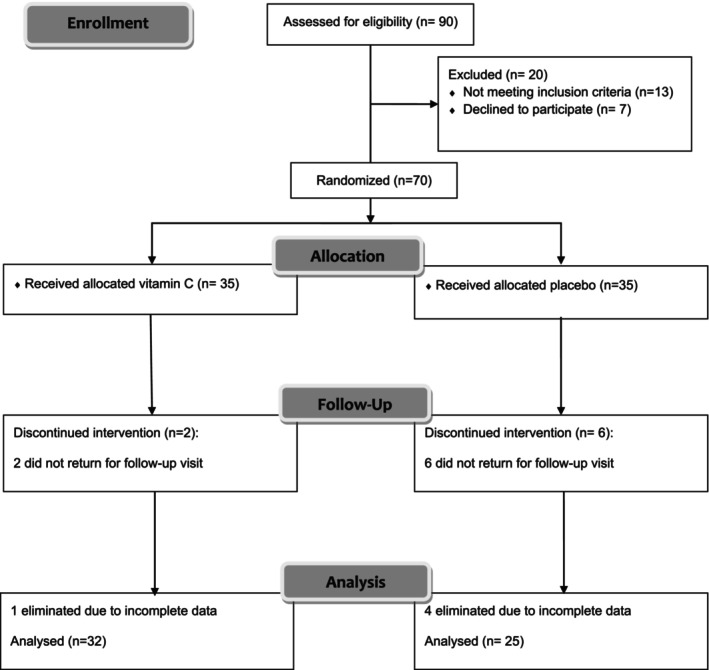
Flow diagram representing case selection for the trial program.

Of the 90 participants assessed for eligibility criteria, 70 patients enrolled with a mean age of 55 years. They were randomly divided into two groups to receive 500 mg/day vitamin C tablets (*n* = 35) or matched placebo (*n* = 35). As Figure 1 shows, six participants in the placebo group and two participants in the vitamin C group did not attend the follow‐up visit, and 62 participants completed the planned 8‐week follow‐up period. Due to incomplete information and missing laboratory evaluation, one patient treated with vitamin C and four treated with the placebo were excluded from the study. Finally, 57 patients with T2D were included in the final analysis (Figure 1). The demographics and anthropometric characteristics of both groups are described in Table 1. There was no significant difference between the two groups regarding the baseline characteristics and diabetes medications, including oral antihyperglycemic medications and insulin use (*p*‐value > .05). Furthermore, the baseline serum levels of inflammatory, oxidative, and antioxidant biomarkers were not significantly different between the two arms of the study (all *p*‐value > .05; Table 2). Therefore, the two groups were relatively matched and comparable.

**TABLE 1:** Baseline characteristics of the study participants.

Variable	Vitamin C (*n* = 32)	Placebo (*n* = 25)	*p*‐value
Gender [Women: Men]	21:11	15:10	.662
Age [years]	54.86 ± 10.0	55.00 ± 12.9	.806
Duration of diabetes [years]	9.57 ± 6.47	9.73 ± 5.28	.920
Waist circumference [cm]	94.45 ± 12.1	91.21 ± 18.1	.441
BMI (kg/m^2^)	27.88 ± 4.22	27.33 ± 4.08	.599
SBP (mmHg)	124 ± 18.6	133 ± 21.7	.228
FPG (mg/dL)	172.0 ± 78.8	161.5 ± 56.0	.564
HbA1c (%)	7.72 ± 2.20	7.77 ± 1.24	.926
Urea (mg/dL)	33.73 ± 13.2	27.09 ± 12.1	.350
Cr (mg/dL)	0.99 ± 0.21	0.91 ± 0.21	.259
eGFR (ml/min)	76.69 ± 14.22	80.10 ± 15.82	.462
Uric acid (mg/dL)	5.51 ± 1.4	4.53 ± 1.1	.094
Serum insulin (mIU/L)	18.91 ± 13.7	19.96 ± 12.4	.749
HOMA‐IR	5.56 (3.27–14.56)	6.78 (4.04–10.75)	.903
Cholesterol (mg/dL)	181.37 ± 38.86	183.90 ± 52.83	.870
LDL (mg/dL)	101.3 ± 33.7	97.58 ± 36.0	.723
HDL (mg/dL)	43 (35–50.5)	45 (39–51)	.357
TG (mg/dL)	192.17 ± 95.2	157.4 ± 66.4	.131
AST (U/L)	18.50 ± 7.93	21.83 ± 12.8	.324
ALT (U/L)	20.65 ± 12.2	23.04 ± 16.8	.636
ox‐LDL (mU/L)	55.08 ± 17.94	50.17 ± 17.86	.308
MDA (μmol/L)	3.78 ± 0.07	3.76 ± 0.09	.414
AGEs (%)	85.0 ± 5.04	84.91 ± 5.20	.946
AOPP (μmol/L)	218.26 ± 15.77	216.94 ± 16.13	.758
FRAP (μmol/L)	923.81 ± 132.47	939.56 ± 129.29	.654
TNF‐α (pg/mL)	894.62 ± 37.16	885.76 ± 34.37	.360
hs‐CRP (mg/L)	15.4 (5.9–45)	12.2 (4.5–31)	.362
Hypoglycemic drugs (%)			
Sulfonylurea *n* (%)	7 (21.9)	4 (16)	.904
Metformin *n* (%)	7 (21.9)	8 (32)	
Sulfonylurea + Metformin *n* (%)	12 (37.5)	8 (32)	
Metformin + Insulin *n* (%)	3 (9.3)	3 (12)	
Insulin *n* (%)	3 (9.3)	2 (8)	

*Note*: Data are presented as either Mean ± SE, frequency, or percent. *p*‐value <.05 is considered significant.

*Abbreviations*: AGEs, advanced glycation end products; ALT, alanine aminotransferase; AOPP, advanced oxidation protein products; AST, aspartate aminotransferase; BMI, body mass index; Cr, Creatinine; eGFR, estimated Glomerular filtration rate; FPG, fasting plasma glucose; FRAP, ferric reducing ability of plasma; HbA1c, hemoglobin A1c; HDL‐C, high‐density lipoprotein cholesterol; HOMA‐IR, Homeostasis model assessment of insulin resistance; hs‐CRP, highly sensitive C‐reactive protein; LDL‐C, low‐density lipoprotein cholesterol; MDA, malondialdehyde; Ox‐LDL, oxidized low‐density lipoprotein; SBP, systolic blood pressure; TG, triglycerides; TNF‐α, tumor necrosis factor α.

**TABLE 2:** A summary of alteration of measured oxidative, antioxidative, and inflammatory biomarkers in trial arms.

Variables	Vitamin C (*n* = 32)				Placebo (*n* = 25)			
	Baseline	Follow‐up	MD (95% CI)	*p*‐value^a^	Baseline	Follow‐up	MD (95% CI)	*p*‐value^b^
Lipid peroxidation								
Ox‐LDL (mU/L)	55.08 ± 17.94	52.90 ± 18.13	2.18 (−4.0–8.4)	.480	50.17 ± 17.86	47.12 ± 14.41	3.04 (−4.04–10.12)	.384
MDA (μmol/L)	3.78 ± 0.07	3.4 ± 0.26	0.38 (0.28–0.47)	**<.001**	3.76 ± 0.09	3.75 ± 0.10	0.00 (−0.01–0.01)	.581
Protein oxidation								
AGEs (%)	85.0 ± 5.04	79.83 ± 7.19	5.16 (2.13–8.20)	**.002**	84.91 ± 5.20	84.93 ± 5.17	0.03 (−0.32–0.25)	.828
AOPP (μmol/L)	218.26 ± 15.77	211.23 ± 27.1	7.02 (−4.78–18.82)	.234	216.94 ± 16.13	216.94 ± 16.07	0.00 (−0.09–0.08)	.924
Inflammatory markers								
hs‐CRP (mg/L)	3.11 ± 3.23	3.15 ± 3.31	0.03 (−5.95–5.25)	.899	2.39 ± 2.64	2.38 ± 2.36	0.01 (−8.89–9.15)	.976
TNF‐α (pg/mL)	894.62 ± 37.16	882.68 ± 78.04	11.94 (−20.14–44.01)	.454	885.76 ± 34.37	885.5 ± 34.22	0.04 (−0.92–0.84)	.926
Antioxidant marker								
FRAP (μmol/L)	923.81 ± 132.47	956.06 ± 124.01	32.25 (−96.20–31.70)	.312	939.56 ± 129.29	939.56 ± 129.7	0.00 (−0.98–0.98)	1.0

*Note*: Data are presented as mean ± SD. Bold values indicate *p* ≤ .05 considered significant.

*Abbreviations*: AGEs, advanced glycation end products; AOPP, advanced oxidation protein products; CI, confidence interval; FRAP, ferric reducing ability of plasma; hs‐CRP, highly sensitive C‐reactive protein; MD, mean difference; MDA, malondialdehyde; Ox‐LDL, oxidized low‐density lipoprotein; TNF‐α, tumor necrosis factor α.

^a^ Comparing baseline and follow‐up measurements within the vitamin C group.

^b^ Comparing baseline and follow‐up measurements within the placebo group.

### 3.2 Outcomes

#### 3.2.1 Within‐group changes in the vitamin C arm

**FIGURE 2:**
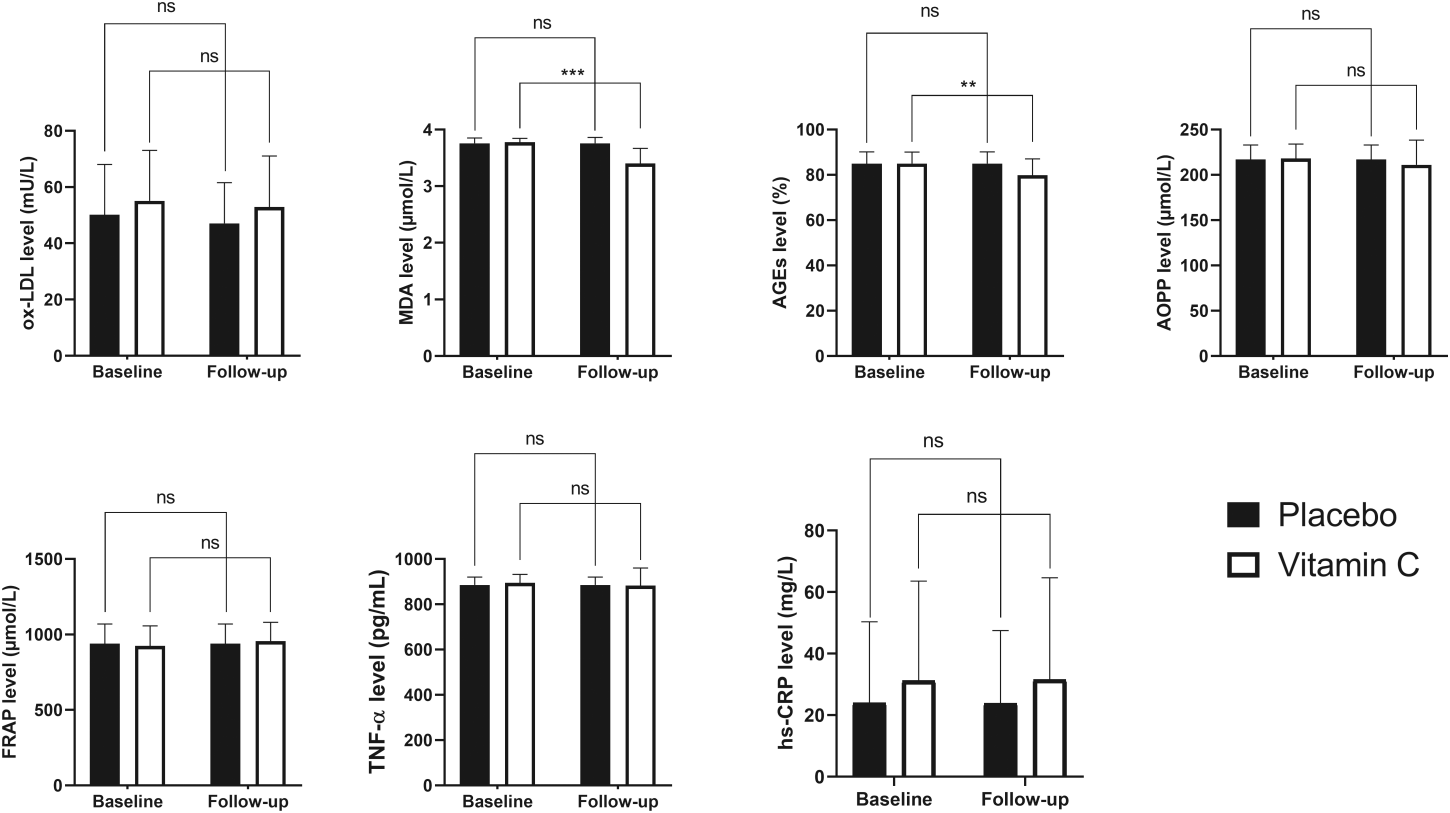
Baseline state and postsupplementation level of oxidative stress, antioxidative and inflammatory biomarkers in the trial groups. ns, not significant; **, .001 < *p*‐value <.01; ***, *p*‐value <.001.

Following 8 weeks of intervention, serum oxidative stress markers, including AGEs and MDA, decreased significantly in the vitamin C group [MDA (μmol/L): mean difference (95% CI) = 0.38 (0.28–0.47), *t* (31) = 7.91, *p*‐value < .001; AGEs (%): mean difference (95% CI) = 5.16 (2.13–8.20), *t* (31) = 3.47, *p*‐value = .002] (Table 2; Figure 2). According to Table 2, there was no significant difference in inflammatory markers (CRP and TNF‐α), FRAP, and ox‐LDL levels in the vitamin C arm (*p*‐values > .05). Also, there were no significant changes in weight, systolic and diastolic blood pressure, waist circumference, FPG, HbA1c, urea, creatinine, uric acid, cholesterol, TG, LDL‐C, HDL‐C, AST, and ALT (*p*‐values > .05).

#### 3.2.2 Within‐group changes in the placebo arm

There were no significant changes in MDA or AGEs levels in the placebo group (*p*‐values were .330 and .616, respectively). In addition, there was no significant difference in inflammatory markers (CRP and TNF‐α), FRAP, and ox‐LDL levels in the placebo arm (*p*‐values>.05; Table 2). No significant changes were observed in weight, systolic and diastolic blood pressure, waist circumference, FPG, Hb A1c, urea, creatinine, uric acid, cholesterol, TG, LDL‐C, HDL‐C, AST, and ALT (*p*‐value > .05).

#### 3.2.3 Comparison of the effectiveness of interventions (between‐group comparisons)

Other baseline characteristic variables were analyzed after 8 weeks to detect possible confounders, and the ANCOVA modeling was performed, as shown in Table 3. After 8 weeks, this study did not observe any significant changes in the following variables: the kidney and liver function tests, lipid profile, HbA1c, and glycemic control (all *p*‐values > .05). Based on the univariate ANCOVA, the difference between the baseline and follow‐up levels of MDA and AGEs remained significant after adjustment for age, BMI, glycemic indicators, and lipid profile. In the final model, after adjusting for the major confounders, 56.0% of the variance observed in MDA and 27.9% in AGEs level were due to vitamin C intake (*p*‐value was <.001 and .007, respectively).

**TABLE 3:** Comparing effects of vitamin C and placebo on oxidative, antioxidant, and inflammatory markers – Analysis of Co‐variance (ANCOVA).

Markers	Model 1		Model 2	Model 3	Model 4	Model 5	Model 6			
	*p*‐value	Effect size (%)	*p*‐value	*p*‐value	*p*‐value	*p*‐value	Adjusted follow‐up mean (95% CI)		Effect size (%)	*p*‐value
							Vitamin C	Placebo		
Ox‐LDL (mU/L)	.377	1.4	.505	.13	.23	.198	48.38 (43.68, 53.08)	50.77 (44.86, 56.67)	5	.180
MDA (μmol/L)	**<.001**	99.4	**<.001**	**<.001**	**<.001**	**<.001**	3.42 (3.33, 3.51(	3.71 (3.60, 3.82(	56	**<.001**
AGEs (%)	**.002**	16.6	**.005**	**.003**	**.008**	**.007**	79.81 (76.88, 82.73(	85.35 (81.75, 88.92(	27.9	**.007**
AOPP (μmol/L)	.309	1.9	.337	.229	.234	.119	209.54 (200.2, 218.8(	219.09 (207.73, 230.46(	6.9	.132
hs‐CRP (mg/L)	.106	4.8	.109	.09	.08	.07	30.76 (23.39, 38.23)	28.44 (19.09, 37.80)	9.4	.070
TNF‐α (pg/mL)	.733	0.2	.75	.719	.595	.667	876.97 (844.19, 909.6(	895.30 (856.95, 933.66(	0.8	.663
FRAP (μmol/L)	.438	1.1	.31	.390	.341	.302	961.40 (910.5, 1012.3)	936.90 (869.93, 989.73(	4.6	.302

*Note*: Variance explained by the use of vitamin C; calculated from partial eta squared. Squared values of 1%, 6%, and 13.8% indicate small, medium, and large effect sizes, respectively. Model 1: Baseline; Model 2: Controlling for age, BP, and BMI; Model 3: Controlling for age, BP, BMI, HbA1C; Model 4: Controlling for age, BP, BMI, CHL, and TG; Model 5: Controlling for age, BP, BMI, HDL, TG, and LDL; Model 6: Controlling for age, BP, waist circumference, HbA1C, TG, and LDL. Bold values indicate *p* ≤ .05 considered significant.

*Abbreviations*: AGEs, advanced glycation end products; AOPP, advanced oxidation protein products; FRAP, ferric reducing ability of plasma; hs‐CRP, highly sensitive C‐reactive protein; MDA, malondialdehyde; Ox‐LDL, oxidized low‐density lipoprotein; TNF‐α, tumor necrosis factor α.

### 3.3 Adverse events

Vitamin C supplements led to an upset stomach during or after eating in four patients and nausea in two patients. However, Fisher's exact test analysis showed no significant difference between the two arms (upset stomach: *p*‐value = .123; nausea: *p*‐value = .499). All mild to moderate adverse events were tolerable.

## 4 DISCUSSION

This trial provides novel information on the beneficial and safe effects of vitamin C added to antiglycemic medications in patients with T2D to reduce serum oxidative stress markers. The significant decrease in oxidative markers levels, including AGEs and MDA, persisted after adjustment for confounders with an effect size of 27.9% and 56%, respectively. However, daily administration of vitamin C could not alter the AOPP, ox‐LDL, and FRAP levels.

Current evidence shows that vitamin C has an inhibitory role in the production of protein oxidation biomarkers such as AOPP and AGEs (Grzebyk & Piwowar, 2016). Also, vitamin C can reduce the escalating interaction between oxidation and glycation, resulting in glycation inhibition (Grzebyk & Piwowar, 2016). Due to the similar structure of vitamin C to glucose, it can be replaced with glucose in response to chemical reactions and prevent the nonenzymatic glycosylation of proteins (Afkhami‐Ardekani & Shojaoddiny‐Ardekani, 2007). To our knowledge, this is the first study to elucidate the effect of vitamin C on protein oxidation in a clinical trial setting in patients with T2D. This study found a significant reduction in AGEs levels while AOPP remained unchanged following 8 weeks of vitamin C administration. In a study on rats with experimental diabetes and periodontitis, Toraman et al. showed that AGE values decreased in the group treated with vitamin C (Toraman et al., 2020). Further research is needed to shed light on the possible reasons for such a finding.

The current study showed heterogeneous results for the efficacy of vitamin C in inhibiting lipid peroxidation; contrary to the significant decrease in MDA serum level, the ox‐LDL level did not change. Similar to our findings, two other studies reported that vitamin C supplementation decreased MDA levels in patients with diabetes (Mazloom et al., 2011; Ruknarong et al., 2021). Also, Mahmoudabadi et al. showed that vitamin C supplementation with a dose of 200 mg per day significantly reduced the serum levels of MDA (Mahmoudabadi & Rahbar, 2014). MDA is an aldehyde produced following lipid peroxidation of polyunsaturated fatty acids (Marrocco et al., 2017). Although MDA is a stable secondary lipid peroxidation product, it tends to react with several biomolecules and reform adducts such as proteins, DNA, and LDL (Ayala et al., 2014). In particular, LDL modification due to oxidation, glycation, and MDA binding leads to ox‐LDL formation (Marrocco et al., 2017). Ox‐LDL has been shown to play an essential role in developing atherosclerosis in T2D by stimulating adaptive immunity (Peluso et al., 2012). Although the pathogenic role of ox‐LDL in diabetes is well established, its use as a biomarker of oxidative stress has been controversial due to “its relatively unclear metabolism pathway, heterogeneity of oxidation products and the low specificity of the antibodies, also the diverse findings obtained depending on the assessment methods” (Frijhoff et al., 2015; Maiolino et al., 2013; Marrocco et al., 2017). Another issue that may explain the present discrepancy in the role of vitamin C in attenuating lipid oxidation is that the participants were patients with prolonged diabetes. In contrast, it has been shown that ox‐LDL level depends on the chronicity of the disease (Nakhjavani et al., 2010).

FRAP measurement reasonably estimates the total antioxidant capacity (Benzie & Devaki, 2018). Reduced FRAP levels have been reported in patients with diabetes (Colas et al., 2010; Singh et al., 2012). In a trial conducted on patients with essential hypertension, administration of vitamin C (1 g/day) in combination with vitamin E (400 units/day) for 8 weeks increased FRAP levels in the treatment arm compared to the placebo arm (Rodrigo et al., 2008). However, the exclusive impact of vitamin C on FRAP levels could not be understood with that study. A trial in males with T2D found that vitamin C and/or E supplementation improved reduced glutathione (GSH) (El‐Aal et al., 2018). The current study evaluated the effects of vitamin C administration on the total antioxidant capacity in patients with T2D, which showed an increasing trend. However, it was not significant. Future studies with larger sample sizes and a more extended follow‐up may provide better insight into the relationship between vitamin C and antioxidant capacity in T2D.

In this study, there were no significant changes in the levels of basic inflammatory biomarkers, including CRP and TNF‐α, after vitamin C supplementation. Vitamin C is considered a molecule with anti‐inflammatory properties as it modulates the binding activity of nuclear factor‐kappa B (NF‐κB) (Ajibade et al., 2017; Carr & Maggini, 2017). However, previous studies have investigated the impact of vitamin C therapy on inflammatory biomarkers in different settings and have reported controversial findings. Similar to our results, Khajehnasiri et al. showed that vitamin C added to omega‐3 could not significantly affect the inflammatory status in patients with depression, compared to omega‐3 alone (Khajehnasiri et al., 2013). In another trial, vitamin C supplementation could not affect the plasma levels of IL‐6 and IL‐10 (Aguilo et al., 2014). In a trial by Tousoulis et al., vitamin C supplementation did not affect TNF‐α, CRP, and IL‐6 (Tousoulis et al., 2007). Nevertheless, one study reported that 1000 mg of vitamin C supplementation for 8 weeks significantly reduced CRP and IL‐6 levels in participants with obesity, hypertension, and/or diabetes (Ellulu et al., 2015).

The concentration of vitamin C in plasma and tissue depends on the amount consumed, bioavailability, and renal excretion (Padayatty & Levine, 2016). The recommended daily vitamin C intake for adults is 75–90 mg (Monsen, 2000). However, because the systemic concentration of vitamin C has been demonstrated to be reduced in diabetes, higher doses should be employed (Pari et al., 2018). A study of different amounts of vitamin C administration showed that 500–5000 mg of vitamin C had no negative impact on natural killer cell activity, apoptosis, or cell cycle (Vojdani et al., 2000). Regarding safety, the frequency of adverse events was not different between the two arms of the study. For a minimum of 3 weeks, 500–2000 mg of vitamin C per day has been prescribed in previous studies (Aguilo et al., 2014; Khajehnasiri et al., 2013). We prescribed 500 mg daily for 8 weeks to ensure better compliance and safety.

Although the present trial had considerable strengths, such as the double‐blind, placebo‐controlled design, rigorous adjustment for confounding variables, and similar baseline characteristics for both arms, several limitations should be noted to prevent overgeneralization of the findings. First, a larger sample size could improve the quality of the results. Second, more than the 8‐week trial period may be required to clarify the long‐term effects of vitamin C supplementation. Third, we measured oxidative and inflammatory markers in plasma to evaluate the systemic benefits of vitamin C. At the same time, it has been shown that this kind of vitamin can also affect the oxidative stress level in other tissues, such as the skeletal muscle of patients with diabetes (Mason et al., 2016). Fourth, methods for measuring AGEs need to be better standardized. Fluorometric methods do not detect nonfluorescent AGEs. ELISA methods have limited specificity and reproducibility. The HPLC method can only be used for AGE molecules with a known structure. This study also did not measure vitamin C circulating levels because there is little evidence to suggest that plasma and tissue levels of vitamin C are connected (Collie et al., 2020). Research on patients with insufficient levels at the beginning of treatment and sufficient levels after treatment would be interesting. Finally, this trial evaluated the beneficial effects of vitamin C on a single‐dose basis. Further studies may clarify the optimal dosage of vitamin C in managing patients with T2D.

The results of this study must be considered preliminary. In summary, adding 8 weeks of vitamin C supplementation to hypoglycemic medications showed significant beneficial effects on MDA and AGEs, which are long‐lived molecules in patients with T2D. Further trials with larger sample sizes and more extended follow‐up periods could clarify the precise effect of vitamin C supplementation therapy on oxidative stress and inflammatory conditions in patients with T2D.

## AUTHOR CONTRIBUTIONS

**Soghra Rabizadeh:** Conceptualization (equal); formal analysis (equal); methodology (equal); writing – original draft (equal). **Firouzeh Heidari:** Formal analysis (equal). **Reza Karimi:** Data curation (equal); investigation (equal). **Armin Rajab:** Data curation (equal); writing – original draft (equal). **Shahram Rahimi‐Dehgolan:** Formal analysis (equal); methodology (equal). **Amirhossein Yadegar:** Data curation (equal); formal analysis (supporting); methodology (supporting); validation (equal); writing – review and editing (lead). **Fatemeh Mohammadi:** Data curation (equal); formal analysis (supporting); methodology (supporting); validation (equal); writing – review and editing (lead). **Hossein Mirmiranpour:** Data curation (equal). **Alireza Esteghamati:** Conceptualization (equal); visualization (equal). **Manouchehr Nakhjavani:** Conceptualization (equal); methodology (lead); project administration (lead); supervision (lead); visualization (lead); writing – original draft (lead); writing – review and editing (lead).

## FUNDING INFORMATION

This study was supported by a grant from the National Institute for Medical Research Development (NIMAD) under award number 971098.

## CONFLICT OF INTEREST STATEMENT

There is no conflict of interest to declare.

## ETHICAL STATEMENTS

All procedures were in accordance with the Helsinki Declaration, and all the procedures involving human subjects/patients were approved by the Ethics Committee of the National Institute for Medical Research Development of Iran (Code of Ethics: IR.NIMAD.REC.1397.123). Written informed consent was obtained from all the participants.

## Supporting information


Appendix S1.

Appendix S2.

## Data Availability

The data that support the findings of this study are available from the corresponding author upon reasonable request.
