# Supplementary material for: Vitamin C supplementation lowers advanced glycation end products (AGEs) and malondialdehyde (MDA) in patients with type 2 diabetes: A randomized, double‐blind, placebo‐controlled clinical trial
Source: Food Sci Nutr. 2023 Jun 30;11(10):5967–77. doi: 10.1002/fsn3.3530 (PMC10563761; doi:10.1002/fsn3.3530)
Supplement: Supplementary file 1 — Appendix S1. [file FSN3-11-5967-s002.docx]

| Exclusion criteria | Reason |
| --- | --- |
| Class III/IV heart failure | Inflammation and oxidative stress-Poor nutrient absorption - The use of diuretics may cause electrolyte imbalances and affect nutrient absorption and excretion [1]. |
| Any history of vascular complications of diabetes or hypertension | Vitamin C metabolism and utilization are affected by oxidative stress and inflammation in these conditions [2]. |
| Any history of chronic disease (including lung, kidney, or liver disease) | When these conditions exist, oxidative stress and inflammation affect vitamin C metabolism [2]. |
| Body mass index more than 35 kg/m^2^ | Vitamin C levels are inversely related to BMI and are lowest in obese individuals [2]. |
| Systolic blood pressure more than 160 mmHg | Oxidative stress and inflammation affect vitamin C metabolism [2]. |
| Diastolic blood pressure more than 90 mmHg | Vitamin C metabolism is affected by oxidative stress and inflammation [2]. |
| Use prescription or over-the-counter vitamins | There is a possibility that it contains vitamin C and thus interferes with the intervention. |
| Smoking history | The level of vitamin C is affected by oxidative stress and oxidants produced by smoking [2]. |
| Alcohol use in the past month | Vitamin C excretion in the urine is increased by alcohol [3]. |
| Aspirin intake in the past year | The absorption of vitamin C may be hindered by aspirin [4]. |
| With or planning pregnancy | Hemodilution and active transfer of vitamin C to the developing fetus lower pregnant women's vitamin C levels [2]. |
| History of corticosteroid intake | Vitamin C preserves corticosteroid levels [5]. |
| History of hypersensitivity to vitamin C | In order to ensure the safety of patients. |
| History of acute or chronic inflammation | The adverse effects of oxidative stress and inflammation in these conditions on vitamin C metabolism [2]. |

1. Grossniklaus, D.A., et al., *Nutrient intake in heart failure patients.* J Cardiovasc Nurs, 2008. **23**(4): p. 357-63.

2. Carr, A.C. and S. Rowe, *Factors Affecting Vitamin C Status and Prevalence of Deficiency: A Global Health Perspective.* Nutrients, 2020. **12**(7).

3. FAIZALLAH, R., et al., *ALCOHOL ENHANCES VITAMIN C EXCRETION IN THE URINE.* Alcohol and Alcoholism, 1986. **21**(1): p. 81-84.

4. Basu, T.K., *Vitamin C-aspirin interactions.* Int J Vitam Nutr Res Suppl, 1982. **23**: p. 83-90.

5. Fogarty, A., et al., *Corticosteroid sparing effects of vitamin C and magnesium in asthma: a randomised trial.* Respiratory Medicine, 2006. **100**(1): p. 174-179.
