# Supplementary material for: Vitamin C supplementation lowers advanced glycation end products (AGEs) and malondialdehyde (MDA) in patients with type 2 diabetes: A randomized, double‐blind, placebo‐controlled clinical trial
Source: Food Sci Nutr. 2023 Jun 30;11(10):5967–77. doi: 10.1002/fsn3.3530 (PMC10563761; doi:10.1002/fsn3.3530)
Supplement: Supplementary file 2 — Appendix S2. [file FSN3-11-5967-s001.doc]

# **Side Effect Checklist**

**Nausea**

**Vomiting**

**Abdominal Pain**

**Heart burn**

**Diarrhea**

**Constipation**

**Dizziness**

**Headache**

**Dry mouth**

**Skin rash**

**Urinary Retention**

**Flank pain**

**Insomnia**

**Increased Appetite**

**Loss of appetite**

**Weight loss/gain**

**Fatigue**

**Itches**

**Sore throat/tongue**

**Nervousness**

**Restlessness**

**Seizure**

**Tremor**

**Flushing**

**Palpitation**

**Other: __________**

**Other: __________**

**Other: __________**
